# Supplementary material for: Arginine Dysregulation and Myocardial Dysfunction in a Mouse Model and Children with Chronic Kidney Disease
Source: Nutrients. 2023 Apr 30;15(9):2162. doi: 10.3390/nu15092162 (PMC10181438; doi:10.3390/nu15092162)
Supplement: Supplementary file 1 [file nutrients-15-02162-s001.zip › nutrients-2299912-supplementary.pdf]

Supplementary Materials

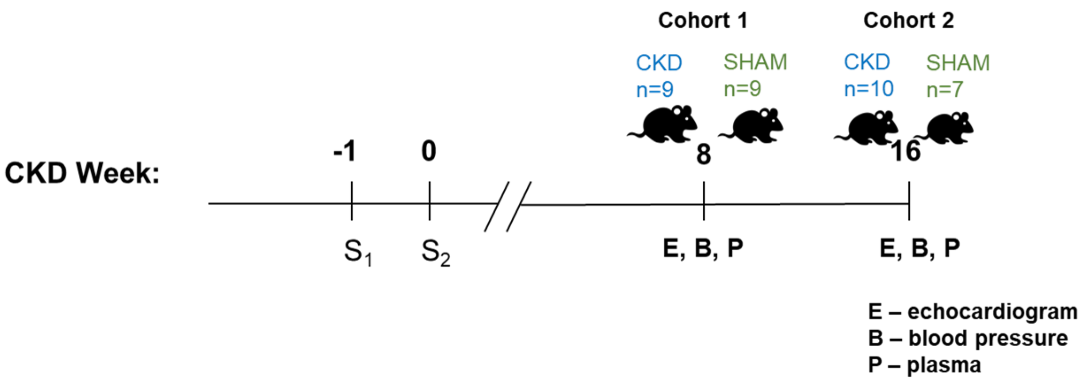

Figure S1. Schematic representing chronic kidney disease (CKD) and control (SHAM) murine experimental plan. Abbreviations: S1, 1st surgery; S2, second surgery CKD, chronic kidney disease.

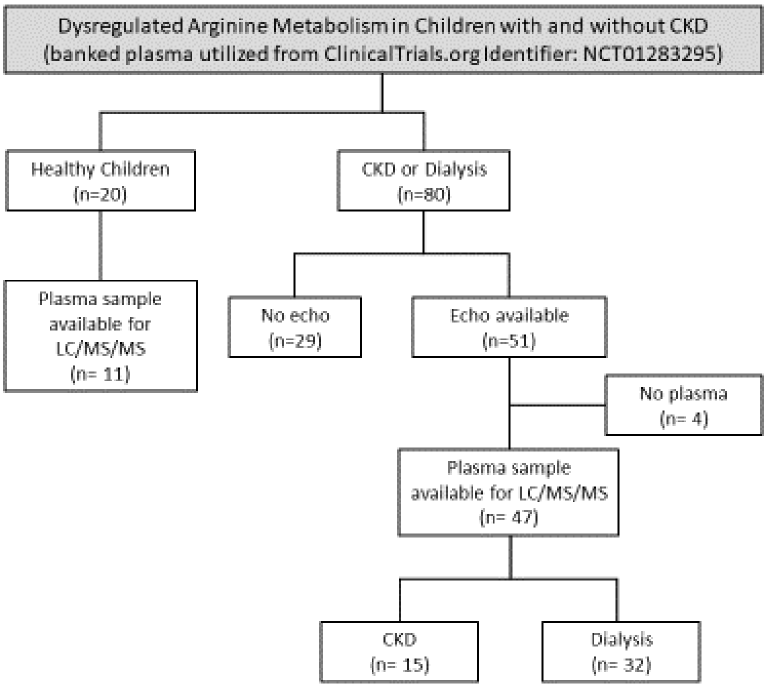

Figure S2. Schematic representing recruitment of banked plasma samples from children with and without chronic kidney disease (CKD)/end-stage renal disease (ESRD) .
